# Socioeconomic factors explain suboptimal adherence to antiretroviral therapy among HIV-infected Australian adults with viral suppression

**DOI:** 10.1371/journal.pone.0174613

**Published:** 2017-04-03

**Authors:** Krista J. Siefried, Limin Mao, Stephen Kerr, Lucette A. Cysique, Thomas M. Gates, John McAllister, Anthony Maynard, John de Wit, Andrew Carr, On behalf of PAART study investigators

**Affiliations:** 1 St Vincent’s Centre for Applied Medical Research, St Vincent’s Hospital, Sydney, New South Wales, Australia; 2 Centre for Social Research in Health, University of New South Wales, Sydney, New South Wales, Australia; 3 Neuroscience Research Australia, University of New South Wales, Sydney, New South Wales Australia; 4 National Association of People with HIV Australia, Sydney, New South Wales, Australia; 5 Department of Interdisciplinary Social Science, Utrecht University, Utrecht, The Netherlands; University of Texas Health Science Center at San Antonio, UNITED STATES

## Abstract

**Background:**

Missing more than one tablet of contemporary antiretroviral therapy (ART) per month increases the risk of virological failure. Recent studies evaluating a comprehensive range of potential risk factors for suboptimal adherence are not available for high-income settings.

**Methods:**

Adults on ART with undetectable viral load (UDVL) were recruited into a national, multi-centre cohort, completing a comprehensive survey assessing demographics, socio-economic indicators, physical health, well-being, life stressors, social supports, HIV disclosure, HIV-related stigma and discrimination, healthcare access, ART regimen, adherence, side effects, costs and treatment beliefs. Baseline data were assessed, and suboptimal adherence was defined as self-reported missing ≥1 ART dose/month over the previous 3-months; associated factors were identified using bivariate and multivariate binary logistic regression.

**Results:**

We assessed 522 participants (494 [94.5%] men, mean age = 50.8 years, median duration UDVL = 3.3 years [IQR = 1.2–6.8]) at 17 sexual health, hospital, and general practice clinics across Australia. Seventy-eight participants (14.9%) reported missing ≥1 dose/month over the previous three months, which was independently associated with: being Australian-born (AOR [adjusted odds ratio] = 2.4 [95%CI = 1.2–4.9], p = 0.014), not being in a relationship (AOR = 3.3 [95%CI = 1.5–7.3], p = 0.004), reaching the “Medicare safety net” (capping annual medical/pharmaceutical costs) (AOR = 2.2 [95%CI = 1.1–4.5], p = 0.024), living in subsidised housing (AOR = 2.5 [95%CI = 1.0–6.2], p = 0.045), receiving home-care services (AOR = 4.4 [95%CI = 1.0–18.8], p = 0.046), HIV community/outreach services linkage (AOR = 2.4 [95%CI = 1.1–5.4], p = 0.033), and starting ART following self-request (AOR = 3.0 [95%CI = 1.3–7.0], p = 0.012).

**Conclusions:**

In this population, 15% reported recent suboptimal ART adherence at levels associated in prospective studies with subsequent virological failure, despite all having an undetectable viral load. Associations were with social/economic/cultural/patient engagement factors, but not ART regimen/clinical factors. These associations may help identify those at higher risk of future virological failure and guide patient education and support.

## Introduction

Antiretroviral therapy (ART) of HIV infection is effective at increasing quality of life and preventing HIV transmission, progression to AIDS, and death [1]. Suppression of HIV viral load to below detectable limits is the key predictor of efficacy [2, 3]. Once ART is initiated, it must be continued daily for life [4]. At least 95% adherence is recommended [5], including for contemporary, single-tablet, once-daily ART regimens [6]. One study comparing two single-tablet, initial ART regimens found that virological suppression at 48 weeks was 88–91% in those with ≥95% adherence, 75–79% with 90–95% adherence, but only 56–70% with <90% adherence [7]. Others have found virological response falls from 89–99% at >95% adherence to 62–75% with ≤95% adherence [6, 8].

Despite its benefits, not all patients fully adhere to ART. Non-adherence encompasses ART interruption as well as ongoing ART use with missed doses. A global meta-analysis estimated 90% ART adherence rates to be only 62% [9]. In Australia, which provides highly subsidised ART to all citizens and permanent residents, an estimated 92% of people on ART achieve viral suppression [10]. However, some patients who achieve virological suppression subsequently experience treatment failure (in Australia, about 3.5% per year [11]).

Australian permanent residents and citizens are able to access subsidised ART through the Pharmaceutical Benefits Scheme. Some patients contribute to the cost of their medicine (a co-payment), with a safety net in place to reduce these costs following meeting the annually-determined threshold. In some circumstances, patients can qualify for a concession card to further reduce their contribution (e.g. social welfare recipients, pensioners, department of veterans’ affairs, etc.). Despite these subsidies, medicine costs in Australia have been noted to be moderate to high in comparison to other OECD countries [12], and even low co-payments have been identified as a potential barrier to accessing medications [13] [14]. Although narrowing, gaps remain in participation in paid employment and other social determinants of health (e.g. education) in HIV-positive adults in Australia [15].

Reasons for suboptimal ART adherence in resource-rich countries in a contemporary setting are not well understood [16]. Much of the literature on adherence pre-dates the era of single tablets regimens, or treatment as prevention [17, 18]. Factors individually associated with lower adherence include complex medication regimens (more than one tablet or dosing time per day) [19, 20], ART toxicity [21], ART costs and financial stress [22], attitudes and beliefs about ART necessity [23, 24], concerns about ART (e.g. side effects, reliance on ART) [24], poor relationships with healthcare providers, sociocultural relationship factors (e.g. unsupportive social networks), substance abuse [25], and HIV-associated neurocognitive disorders [26].

Prior studies of covariates of suboptimal ART adherence have several limitations. Many were not performed in the current era of simple, once-daily ART. Also, to the best of our knowledge, no study has comprehensively assessed the wide range of factors that may influence ART adherence, so it is unknown to what extent various factors are independently associated with adherence, nor which might be most associated. Furthermore, some potentially critical covariates have been studied infrequently. For example, an EMBASE / OVID search of the literature published between 2010 and 2016, with keywords (exploded) “HIV”, “antiretroviral therapy”, and “adherence” limited to adults and available in English language returned 291 results; adding “finance/finances/financial”, returned two publications in the OECD context; including previous work completed by our group which did not evaluate an extensive range of variables [22]. We identified only one other study that reported the association between adherence and costs of ART to patients in a resource-rich setting [27]. Furthermore, most of the available literature in the OECD setting reports on findings from the United States, which may not be generalizable to the Australian demographic.

To increase our understanding of adherence behaviours, we conducted a cross-sectional analysis of the baseline sample, investigating a large number of patient, treatment, and socio-economic characteristics to determine the extent and predictors of suboptimal ART adherence in HIV-infected adults in Australia despite a suppressed viral load.

## Methods

### Study design and setting

We established a national, 2-year cohort study of HIV-infected adults on ART with an undetectable viral load. Participants were enrolled at 17 Australian general practice, sexual health, and hospital outpatient clinics between September 2013 and November 2015.

Ethical approval was obtained from the Human Research Ethics Committee at each study site. Written, informed consent was obtained from each participant prior to any study activity. Approving HREC names and approval reference numbers are: St Vincent’s Hospital HREC (HREC/12/SVH/186), ACT Health Canberra HREC (ETH.7.13.178), Government of Western Australia South Metropolitan Health Service HREC (ref 13/70), The Alfred Hospital HREC (444/14) and Monash Health HREC (15O28X).

To avoid selection bias (e.g. selecting only those patients who were compliant and would complete the questionnaire), site co-ordinators (usually a research nurse) were instructed to invite all eligible patients during routine clinics, until each site reached its predetermined sample size (proportional to its estimated eligible patient population and capacity to recruit). The aim was to recruit a representative sample of patients at each site and, to not exclude patients deemed at risk of ART failure for any reason. This allowed all patients seen at the study sites over the recruitment period an equal opportunity of participation in the cohort.

### Participants

HIV-infected adults (≥18 years of age) were eligible if they were on ART, had an HIV viral load less than 50 copies/mL plasma (at enrolment or on most recent assessment), could complete the study questionnaire (with the assistance of an interpreter, if required), and had HIV viral load, CD4 T-lymphocyte count, full blood count and biochemistry results in the last 3 months.

### Assessments

#### Questionnaire

Using a study-provided laptop computer, participants complete a 204-question questionnaire (divided between 124 items and 13 themes) at baseline, Month 12, and Month 24 (this report focuses on the baseline data only). Questionnaire feasibility had been tested in a pilot study, which found a mean completion time of 40 minutes with 99% data completion [28].

The annual patient self-completed questionnaire incorporates a series of measures [22, 29–39], assessing the following categories (themes) of variables: socio-demographic characteristics (18 items), financial and employment status (8 items), income (2 items), healthcare and treatment access (19 items), physical health (5 items), mental health (2 items), quality of life (1 item), drug and alcohol use (19 items), life stressors and social supports (3 items), HIV disclosure and perceived or experienced stigma (5 items), ART regimen, side effects, consistent use, and dose adherence (24 items), ART-related necessity beliefs and concerns (10 items), non-ART medication (8 items). Table 1 includes the detailed measures, the majority either existing (e.g. SMAQ [38], PHQ-9 [35] [36], CAGE [39], see Table 1) or pre-validated (e.g. the majority of those have been used among HIV-positive Australians, see Table 1). From the 43-item PROQOL-HIV scale [37]), 41 items were maintained, and two original items (general health and the impact of ART-related side-effects on treatment adherence items) were replaced by separate questions on general health (without the limitation of the past two-week timeframe) and a more detailed set of questions on ART side effects in our study.

**Table 1:** Questionnaire composition.

Heading	Number of Items	Item clusters	Embedded items / literature source
Demographics	18	age, sex, sexual orientation, country of birth, citizenship status, English fluency, Medicare status, partner, household, education	Positive Health (ph) Cohort [29]
Physical health	5	prior AIDS, hospitalizations, time in bed / off work due to illness	Positive Health (ph) Cohort [29]
Mental health / emotional wellbeing	22	mood, alcohol and illicit drug use	Patient Health Questionnaire (PHQ-9) [35, 36], Professional Quality of Life Scale (the PROQOL-HIV scale) [37], CAGE [39] questionnaire for alcohol dependence
Life stressors, social support	3	Events, severity, social network	The Positive Health (ph) Cohort [29]
HIV disclosure, stigma	5	Disclosure, stigma, discrimination	National Association of People With HIV Australia (NAPWHA) HIV Stigma Audit’s Community Survey [34], Professional Quality of Life Scale (the PROQOL-HIV scale)[37]
Health and treatment perception	10	Attitudes to ART and HIV, adverse events, mood	Chronic Illness Acceptance Questionnaire [33], Pleasure and Sexual Health (PASH) study [32]; Beliefs About Medications Scales (BMQ-HAART) [31]
HIV healthcare access, treatment adherence, side effects	51	Current / prior ART and concomitant medications: doses and pills / day, dosing requirements, side effects, adherence to treatments doctors and allied health professional: frequency of attendance, changes to healthcare team, costs of care	Simplified Medication Adherence Questionnaire (SMAQ) [38],
Financial and employment status	10	Income, life costs, healthcare costs	St Vincent’s Immunology B Ambulatory Care’s finance and adherence survey [22]

Participants were asked if they had met the Medicare Safety Net threshold, this threshold provides financial relief for medical services covered under the Medicare benefits system (e.g. a discounted patient co-payment for prescribed pharmaceuticals). In 2015, the Medicare safety net threshold was A$2,000 for a regular Medicare holder, and A$638.40 for a concessional status cardholder [40].

#### Neurocognitive assessment

For the present analysis, neurocognitive function was assessed at baseline using computerized screening (Cogstate brief battery [41]) (future analyses will include data from Months 12 and 24). The Cogstate brief battery includes four tasks (detection, identification, one back and the one-card learning tasks) examining: processing speed, sustained attention, divided attention, visual learning and working memory [41]; and has previously been validated as a reliable screening tool in HIV-positive adults similar to the currently enrolled cohort [42]. Z-scores for each of four domains were generated using Cogstate normative standards [42] and were used to calculate a global deficit score (GDS) [43, 44]. Adjustments were not made for depression or substance use because neither correlated with GDS in this sample (p = 0.122 and p = 0.63 respectively) [45]. The standard GDS of >0.5 (averaged over four tasks [43]) was used as the cut-off point for cognitive impairment, and was analysed using the dichotomous classification of impaired or unimpaired.

#### Antiretroviral dispensing records

ART dispensing data for the 12 months preceding the baseline assessment were collected from every ART-dispensing pharmacy serving each study participant. Data collected are: antiretroviral medications dispensed, months dispensed, total cost to patient (inclusive of any patient co-payment), and whether medication(s) were obtained via a clinical trial.

#### Clinical and laboratory data

The study coordinator collects clinical and laboratory data semi-annually for the duration of follow-up including: medical history; HIV history (date and mode of transmission, past AIDS-defining illnesses, opportunistic infections, serious non-AIDS events [46]); ART history (date of commencement, components and duration of current regimen, pill burden, dosing frequency and requirements, duration of undetectable HIV viral load); concomitant medications; comorbidities; sexually transmitted infections; and patient retention in care (number of attendances, missed appointments, clinical trial enrolment, directly-observed therapy). Laboratory data collected are: HIV viral load, CD4+ T-lymphocyte counts (current and nadir), haemoglobin, estimated glomerular filtration rate (eGFR) and alanine aminotransaminase (ALT).

### Outcome variable

The main outcome variable for the present analysis of the baseline data was ART adherence over the previous three months as reported by participants at study entry. Given the cohort design, all participants included at baseline had an undetectable viral load within three months prior to enrolment. As the main study objective is to identify risk for adherence over time, the cohort data will be analysed in future (when data become available), for participants who experience ART failure. For the present analysis, we were interested in ascertaining the actual adherence of this virologically controlled sample. Given the requirement of >95% adherence to ART, even in contemporary regimens, missing greater than one dose per month places a patient at risk of virological failure. Therefore, suboptimal adherence was defined as an average of at least 1 missed ART dose per month over the previous three months [6, 7], based on participant self-report. This was ascertained by asking participants how many ART doses were missed/skipped in the previous 3-months.

Participants taking once-daily ART (by single tablet regimen or otherwise) who missed one ART dose per month or more were considered to have had suboptimal adherence. Those participants who took twice-daily ART and missed doses at a threshold of >2 doses per month for three months were considered sub-optimally adherent. For the 23 participants taking twice-daily ART who missed less than two doses per month for three months (but missed more than one dose per month; e.g. missing 5 doses over the 3-month period), additional sensitivity analysis was conducted.

### Sample size

The main study was powered based on a previous study conducted by our group that showed 9% of patients had ceased or interrupted medication, and 29% of those who had difficulty meeting pharmacy costs had ceased medication [22]. Assuming characteristics associated with non-adherence would be more prevalent in the non-adherent group, a sample size of 500 was sufficient to give 85% power to detect an odds ratio ≥3 for associations with a covariate, with a prevalence of 29% in the sub-optimally adherent versus adherent participants, at a 2-sided significance level of 5%.

### Statistical methods

For items that were part of a validated scale, results were used to generate a summary score as intended (e.g. PHQ9 for depression [35], PROQOL-HIV [37] and CAGE for alcohol abuse [39]). Categorical data were re-coded into binary responses (e.g. housing: subsidized or not). Continuous data were dichotomized based on clinical significance where supported (e.g. ALT, eGFR) or on sample median or mean values, depending on distribution (e.g., median/mean split). Those continuous data dichotomised by sample median / mean included: age, income, duration of care from primary HIV physician, cost of healthcare / alternative healthcare services over previous 12-months, money spent last time HIV medication was obtained, money spent in previous 3-months on non-HIV medications, cost of all healthcare needs, CD4 T-lymphocyte cell count, missed clinical appointments, number of life stressors, and concomitant medication pill burden. Data dichotomised by a reference point included: year HIV diagnosed (prior to 1996), nadir CD4 (<200), length of undetectable HIV viral load (>12 months), ART year commenced (prior to 2004), haemoglobin (<130g/L males, <120 g/L females), ALT (>40 U/L males, >35 U/L females), eGFR (<90), hospitalisations / inpatient days (>1), bed days due to illness (>1), doctors’ visits due to illness (>1).

Bivariate associations between adherence and all covariates were assessed. Where individual items were correlated, we selected the one showing the most significant relationship with the outcome variable to avoid multi-collinearity. After assessing all covariates for significance, all of those variables significantly (p<0.05) associated with suboptimal adherence in bivariate analysis were included in a multivariable logistic regression analysis using the Enter method, whereby all variables are entered (forced) into the equation simultaneously.

The multivariable logistic regression methodology was chosen to help explore specific covariates that were associated with suboptimal adherence, rather than using data reduction to identify thematic associations, as a goal of the present analysis was to provide clinicians with specific variables to help identify less adherent patients.

Sensitivity analyses were performed using backward-stepwise and forward-stepwise approaches. Additional sensitivity analysis was conducted after reclassifying those on twice-daily ART dosing who potentially missed less than 2 doses of ART per month in the previous 3 months (e.g. missing 5 doses over the previous three months) from the non-adherent to the adherent group.

All statistical analyses were conducted in IBM SPSS Statistics for Windows, Version 22.0.

## Results

### Main demographic and clinical characteristics

Of the 523 participants enrolled, one (0.2%) did not respond to the primary outcome measure and was excluded from this analysis. Of the 522 participants analysed, 203 (38.9%) were enrolled at sexual health clinics, 174 (33.3%) at hospital clinics, and 145 (27.8%) at HIV high-caseload general practices (sample characteristics are outlined in Table 2). Four-hundred-ninety-four (94.6%) participants were male, and participants had a mean age of 50.8 (SD 12.3) years. Three-hundred twenty-two participants (61.6%) were Australian-born. Two-hundred twenty-six (43.3%) participants were in a relationship, of which 136 (26.1%) were with HIV serodiscordant partners.

**Table 2:** Sample characteristics.

Variables	n (%) or mean (SD)
**Demographic characteristics**	
Age (years; mean, SD)	50.8 (12.3)
Gender (Male)	494 (94.6)
Men who have sex with men	410 (78.5)
Australian born	322 (61.6)
Living alone	212 (40.5)
Speaks English at home	493 (94.3)
Ability to read, speak and understand English rated as ‘excellent’	427 (81.6)
Australian citizen	461 (88.1)
Has Medicare access	508 (97.1)
Met the Medicare safety net^a^ in previous 12-months	94 (18.0)
Has private health insurance	221 (42.3)
Lives in a major city	452 (86.4)
University educated	197 (38.0)
**Financial / employment status**	
On social welfare	212 (40.6)
Required financial assistance in previous 12 months	138 (26.4)
No employment	226 (43.2)
Underemployment (would increase hours if available)	220 (42.1)
Current weekly income after tax (median, IQR)	645 (580)
Lives in public-subsidized accommodation	105 (20.1)
Lives with someone who is financially dependent on them	48 (9.2)
Received financial assistance in previous 12 months	
from family	42 (21.8)
from Centrelink^b^	97 (50.3)
from partner	18 (9.3)
from non-governmental organization	80 (41.4)
In previous 12 months, for financial reasons, had to forego food, groceries, rent, household bills, furniture, clothing, white goods	114 (21.8)
**HIV healthcare and treatment access**	
Uses the following for HIV management:	
hospital based HIV clinic	254 (48.7)
health center specialized in HIV treatment	135 (25.9)
community based general practice	174 (33.3)
sexual health clinic / center	168 (32.2)
naturopath	26 (5.0)
hospital pharmacy	259 (49.6)
home or community care	14 (2.3)
drug or alcohol services	9 (1.7)
HIV-related community organizations or support groups	77 (14.8)
Primary HIV physician	
general practitioner	181 (34.7)
hospital physician	223 (42.7)
sexual health physician	114 (21.8)
Study enrolment site	
high-caseload general practice	145 (27.8)
hospital located clinic	174 (33.3)
sexual health clinic / center	203 (38.9)
Feels actively involved in the management / treatment of HIV	506 (96.9)
Consults with primary HIV physician at least every six months	512 (98.1)
Duration of care from primary HIV physician (years: mean, SD)	11.3 (8.0)
Changed primary HIV physician in previous 12 months	80 (15.3)
No payment required from patient for HIV consultations	288 (82.8)
Has seen other medical specialists in the previous 12 months	321 (61.5)
Has other healthcare providers involved in HIV care	324 (62.1)
Cost of healthcare services over previous 12 months (dollars: mean, SD)	281 (616)
Cost of alternative healthcare services over previous 12 months (dollars: mean, SD)	675 (1161)
Cost a barrier to accessing medical services in the previous 12 months	71 (13.6)
ART pharmacy charges a patient co-payment	292 (55.8)
Money spent last time HIV medication obtained (dollars: mean, SD)	106 (410)
Money spent in previous three months on non-HIV medication (dollars: mean, SD)	145 (434)
Cost of all health needs (except medications) in previous 3 months (dollars: mean, SD)	303 (1135)
**HIV history**	
HIV diagnosed prior to 1996	213 (40.8)
Male-to-male sexual transmission of HIV	406 (77.6)
Nadir CD4 T-lymphocyte count <200 cells	202 (38.7)
Previous AIDS	120 (22.9)
**Comorbidities**	
Heart disease	57 (10.9)
Hypertension	94 (18.0)
Stroke	9 (1.7)
Peripheral vascular disease	8 (1.50)
Diabetes	31 (5.9)
Chronic liver failure	2 (0.4)
Chronic kidney disease	14 (2.7)
Other diagnosed comorbidity	102 (19.5)
**Current health**	
CD4 T-lymphocyte count (mean, SD)	659 (273)
Length of undetectable HIV viral load >1 year	399 (76.4)
Currently enrolled on a clinical trial	45 (8.6)
Anaemia^c^	33 (6.3)
Elevated ALT^d^ (>40 U/L males, >35 U/L females)	130 (24.9)
eGFR^e^ <90 mls/min/1.73m2	290 (55.6)
Hepatitis co-infection	70 (13.4)
Sexually transmitted infection in previous 12-months	71 (13.6)
Hospitalized for ≥1 night in previous 12 months	108 (20.7)
Missed ≥1 clinic appointment in previous 12 months	71 (13.6)
**Physical health**	
Self-reported good / very good overall health	435 (83.3)
One or more bed days due to illness in previous 12 months	284 (54.4)
One or more doctor visits due to illness in previous 12 months	358 (68.6)
Greater than one hospital inpatient day in previous 12 months	100 (19.2)
**Mental health**	
Major depressive disorder on PHQ-9[35, 36]	87 (16.7)
Psychiatric illness—currently clinically active	112 (24.3)
**Cognitive function**	
Neurocognitive impairment	148 (28.3)
**Alcohol and drug use**	
Alcohol dependent (CAGE[39])	106 (20.3)
Use of following drugs monthly or more in previous 12 months:	
cigarettes	143 (27.4)
marijuana / hash	94 (18.0)
amyl / poppers	67 (12.8)
benzodiazepines	39 (7.5)
ecstasy	1 (0.2)
injected speed / amphetamines	20 (3.8)
snorted or smoked speed / amphetamines	17 (3.3)
injected cocaine	2 (0.4)
snorted cocaine	3 (0.6)
crystal methamphetamine	23 (4.4)
GHB / GBH / liquid E / fantasy	6 (1.1)
LSD	2 (0.4)
PDE5 inhibitor (“viagra” or ‘similar’)	67 (12.8)
heroin	3 (0.6)
methadone	9 (1.7)
opiates	11 (2.1)
**Life stressors**	
More than 2 major stress events in previous 12 months	133 (25.5)
**Social support**	
Married / de facto / in regular relationship	226 (43.2)
In a serodiscordant sexual relationship	136 (26.0)
Received less social support than wanted / needed	302 (57.9)
Not linked to an HIV support organization	388 (74.3)
**HIV disclosure, stigma and discrimination**	
Did not disclose HIV status	25 (4.8)
Has been made to feel ashamed of HIV diagnosis	224 (43.0)
Has felt blamed for having HIV	173 (33.1)
Has felt avoided, excluded or rejected for having HIV	214 (41.0)
Has had awkward interactions for having HIV	248 (47.5)
**Antiretroviral therapy**	
ART as a single tablet regimen	158 (30.3)
Once-daily ART dosing	333 (63.7)
Twice-daily ART dosing	186 (35.6)
ART requires fasting / food conditions	351 (67.2)
Commenced ART within one year of diagnosis	245 (46.8)
Commenced ART prior to 2004	247 (47.3)
Receiving ART through a clinical trial	28 (5.4)
Receiving ART through directly observed therapy (DOT)	8 (1.5)
When started ART felt ‘not at all’ / ‘only somewhat’ informed about ART	
side effects	178 (34.1)
benefits	115 (22.0)
dosing requirements	44 (8.4)
lifestyle impacts	151 (28.9)
own ART regimen	106 (20.3)
Reason for starting ART:	
to prevent HIV disease progression to AIDS	325 (62.6)
to reduce HIV symptoms	167 (32.2)
to prevent transmission to HIV-negative partners	101 (19.5)
to prevent transmission to the community	94 (18.1)
due to high viral load	233 (44.9)
due to low CD4 t-lymphocyte cell count	263 (50.7)
following doctor’s advice	328 (63.2)
following their own request	64 (12.3)
Never speaks with HIV doctors or nurses about	
balancing ART regimen with overall health and lifestyle	174 (33.5)
side effects associated with ART	101 (19.5)
delaying, interrupting or changing ART	250 (48.3)
cost burden of ART	425 (82.1)
I forget to take ART medications	242 (46.4)
I am careless at times about taking ART medications	86 (16.5)
Sometimes if I feel worse I stop taking ART medications	48 (9.2)
Skipped ART once or more in the previous weekend	35 (6.7)
Experienced ART side effects in the previous 12 months	297 (56.9)
Experienced difficulties accessing pharmacy for ART	32 (6.1)
Missed ART once or more in the previous week	83 (15.9)
Delayed / interrupted ART in the previous 12 months	34 (6.5)
Delayed / interrupted ART in the previous 12 months for ≥1 week	20 (4.0)
Doctor unaware of ART interruption / delay in previous 12 months	7 (1.3)
Delayed / interrupted ART prior to 12 months ago	85 (17.5)
Delayed / interrupted ART prior to 12 months ago for ≥1 week	66 (12.6)
Doctor unaware of prior ART interruption / delay	14 (2.7)
**ART necessity concerns**	
Necessity concerns summary score^f^ (mean, SD)	62.0 (7.0)
**Concomitant medications**	
Daily concomitant medication pill burden (mean, SD)	3.6 (4.3)
Delayed / interrupted in previous 12 months	60 (14.0)
Delayed / interrupted in previous 12 months for ≥1 week	37 (7.1)
Doctor unaware of interruption / delay in previous 12 months	27 (5.2)
Delayed / interrupted prior to 12 months ago	49 (12.3)
Delayed / interrupted prior to 12 months ago for ≥1 week	32 (6.1)
Doctor unaware of prior ART interruption / delay	19 (3.6)
**PRO-QOL HIV**	
PRO-QOL HIV summary score^g^ (mean, SD)	40.1 (23.4)

^a^ whereby medical costs—including pharmaceutical co-payments, are capped after reaching an annual threshold

^b^ social services

^c^ Anaemia defined by haemoglobin <130g/L in men and <120g/L in women[47]

^d^ ALT = alanine aminotransaminase

^e^ eGFR = estimated glomerular filtration rate

^f^ sample summary score (mean) (higher scores indicative of less belief in necessity or acceptability of ART)

^g^ sample summary score (mean) (higher score indicative of lower quality of life)

Mean CD4+ T-lymphocyte count was 659 cells/mm^3^ (SD 273), 122 (22.9%) had a prior AIDS-defining illness, and 70 (13.4%) had hepatitis B and/or C co-infection. Eighty-seven (16.7%) participants had symptoms consistent with a major depressive disorder and 148 (28.3%) were classified as neurocognitively impaired.

The median weekly after-tax income (including social welfare payments) was $580 Australian dollars (about US$422; €377), and median annualized healthcare expenditure was A$598. Nearly all participants (507 [97.1%]) had Medicare coverage (publically-funded hospital and medical services), and 94 (18.0%) had reached the “Medicare safety net” in the previous 12 months. One-hundred thirty-eight (26.4%) participants had relied upon financial assistance to obtain basic necessities (e.g. rent, groceries, utilities) over the previous 12 months, and 114 (21.8%) participants had insufficient financial means to meet basic necessities over the previous 12 months. One-hundred-four (19.9%) participants lived in subsidized housing and 212 (40.6%) were on social welfare.

Seventy-seven (14.8%) participants were linked with one or more HIV community organizations / peer support groups as part of their HIV care, and 14 (2.3%) were receiving home-care services.

### ART regimen and adherence

The median duration of undetectable HIV viral load was 3.3 years (IQR 1.2–6.8 years), and the median ART duration was 11.0 years (IQR 5–19 years), with 137 (26.3%) participants on their current ART regimen for no more than 12 months. Three-hundred thirty-three participants (63.8%) were taking once-daily ART and 158 (30.3%) were taking a single-tablet ART regimen; 352 participants (67.4%) were on regimens with specific food / fasting requirements. Two-hundred eleven (40.4%) participants were on a non-nucleoside reverse-transcriptase inhibitor (NNRTI) and nucleoside / nucleotide reverse transcriptase inhibitor (NRTI/NtRTI) regimen, 99 (19.0%) were on an NRTI plus integrase strand transfer inhibitor (INSTI) regimen, 79 (15.1%) were on an NRTI plus protease inhibitor (PI) regimen, and 133 (25.5%) were on an alternative regimen (predominantly 3-class or NRTI-sparing). Three-hundred twenty-eight participants (62.8%) had started ART following advice from their treating doctor, while 64 participants (12.3%) had started ART following their own request; 195 participants (37.4%) had started treatment to prevent transmission to partners or their community.

Seventy-eight participants (14.9%) reported missing an average of at least one ART dose per month over the previous 3 months (Table 3). Associations with covariates in bivariate analyses are shown in Table 4. The multivariable logistic regression model was statistically significant (x^2^ = 123(45), p<0.001) and correctly classified 86.6% of cases. The final model contained 501 participants with 100% data collected on all included variables. Suboptimal ART adherence was independently associated with the following variables (Table 5): being born in Australia; not being in a relationship; having reached the Medicare safety net threshold; living in subsidized housing; receiving home-care services; linkage to HIV community organizations; and having started ART at the patient’s request. Those with more than three of these variables present had a particularly elevated rate of suboptimal ART adherence (Table 6).

**Table 3:** Self-reported ART adherence.

	Yes	No
	n (%)	n (%)
Missed ≥3 doses in previous 3 months	78 (14.9)	444 (85.1)
Delayed / interrupted ART for ≥1 week in the previous 12 months	20 (3.8)	502 (96.2)
Ever delayed / interrupted ART (more than 12-months ago)*	85 (16.3)	401 (76.8)

*36 (6.9%) participant data missing / not applicable (e.g. not on ART prior to 12-months ago)

**Table 4:** Variables associated with suboptimal ART adherence.

Variables		Missed ART in last 3 months		OR^1^	95% CI^2^	P-value
		≥3 (n = 78)n	<3 (n = 444) n			
**Socio-demographic**						
Not currently in a relationship	No	17	207			
	Yes	61	237	3.1	1.8–5.5	<0.001
Born in Australia	No	15	186			
	Yes	63	258	3.0	1.7–5.5	<0.001
Living alone	No	35	276			
	Yes	43	168	2.0	1.2–3.3	0.004
Living in subsidized housing	No	56	362			
	Yes	22	82	1.7	1.0–3.0	0.047
Living outside of an urban area	No	62	391			
	Yes	16	53	1.9	1.0–3.5	0.039
HIV transmission route other than male-to-male sexual intercourse	No	57	370			
	Yes	21	74	1.9	1.1–3.2	0.030
**Finances and employment**						
Medicare safety net* reached in previous 12 months	No	49	343			
	Yes	29	101	2.1	1.2–3.7	0.009
Having no private health insurance	No	22	199			
	Yes	56	245	2.1	1.2–3.5	0.006
Income ≤$580 per week	No	28	233			
	Yes	50	211	2.0	1.2–3.2	0.007
Unemployed	No	36	260			
	Yes	42	184	1.8	1.1–2.9	0.019
Required financial assistance in the previous 12 months	No	39	346			
	Yes	39	98	3.5	2.1–5.8	<0.001
Going without for financial reasons (food, rent, etc.)	No	43	365			
	Yes	35	79	3.8	2.3–6.3	<0.001
Cost was a barrier to accessing HIV healthcare	No	61	390			
	Yes	17	54	2	1.1–3.7	0.022
ART pharmacy expenditure </ = sample mean monthly ART expenditure	No	22	187			
	Yes	56	257	1.9	1.1–3.1	0.021
**HIV healthcare and treatment access**						
Primary HIV care provided by a general practitioner	No	44	304			
	Yes	34	140	1.7	1.0–2.7	0.037
Receives home care services	No	70	438			
	Yes	8	6	8.3	2.8–24.8	<0.001
Receives drug and alcohol services	No	74	439			
	Yes	4	5	4.7	1.2–18.1	0.012
Linked to HIV community organisation(s)	No	56	389			
	Yes	22	55	2.8	1.6–4.9	<0.001
Required other healthcare specialists in previous 12 months	No	21	180			
	Yes	57	264	1.9	1.1–3.2	0.023
Other healthcare providers were involved in HIV management	No	16	182			
	Yes	62	262	2.7	1.5–4.8	0.001
Having difficulty with ART pharmacy access	No	67	423			
	Yes	11	21	3.3	1.5–7.2	0.001
**Physical health**						
Self-rated health status as poor/very poor	No	56	378			
	Yes	22	66	2.3	1.3–3.9	0.004
>1 bed days for illness in previous 12 months	No	23	215			
	Yes	55	229	2.5	1.5–4.4	0.001
≥1 unscheduled doctor visits	No	17	147			
	Yes	61	297	1.8	1.0–3.3	0.045
≥1 overnight hospitalization in previous 12 months	No	54	360			
	Yes	24	84	1.9	1.1–3.3	0.017
Hepatitis co-infection	No	61	393			
	Yes	17	51	2.1	1.2–4.0	0.013
HIV diagnosis prior to 1996	No	38	271			
	Yes	40	173	1.7	1.0–2.7	0.034
**Mental Health**						
Major depressive disorder	No	49	386			
	Yes	29	58	3.9	2.3–6.7	<0.001
**Alcohol and drug use (at least monthly)**						
Cigarette smoking	No	42	337			
	Yes	36	107	2.7	1.6–4.4	<0.001
Marijuana	No	52	376			
	Yes	26	68	2.8	1.6–4.7	<0.001
Benzodiazepine	No	63	420			
	Yes	15	24	4.2	2.1–8.4	<0.001
Injected speed	No	70	432			
	Yes	8	12	4.1	1.6–10.4	0.001
Injected methamphetamines	No	71	428			
	Yes	7	16	2.6	1.0–6.6	0.033
Opiate use	No	74	437			
	Yes	4	7	3.4	1.0–11.8	0.044
**Life stressors**						
Major stressful event in the previous 12 months	No	20	211			
	Yes	58	233	2.6	1.5–4.5	<0.001
**HIV disclosure and stigma**						
Have felt shamed/excluded /having awkward interactions since HIV diagnosis	No	21	197			
	Yes	57	247	2.2	1.3–3.7	0.004
**ART regimen, side effects, consistent use and dose adherence**						
ART initiation prior to 2004	No	30	245			
	Yes	48	199	2.1	1.3–3.4	0.004
ART side effects	No	25	200			
	Yes	53	244	1.7	1.0–2.9	0.033
On a multiple-tablet ART regimen	No	15	143			
	Yes	63	301	2.0	1.1–3.6	0.021
Greater than once-daily ART dosing	No	40	295			
	Yes	38	149	1.8	1.2–3.1	0.010
Feeling uninformed about ART when starting	No	65	414			
	Yes	13	30	2.7	1.4–5.5	0.004
Starting ART following patient request	No	61	397			
	Yes	17	47	2.3	1.3–4.3	0.006
**ART-related necessity concerns**						
Necessity concerns score > sample mean	No	30	247			
	Yes	48	197	2	1.2–3.3	0.005
**Non-ART medication consistent use and adherence**						
Concomitant medication pill burden >2 pills per day	No	31	249			
	Yes	47	195	1.9	1.2–3.2	0.008
**Quality of Life**						
PRO-QOL HIV score > sample mean	No	27	271			
	Yes	51	173	3	1.8–4.9	<0.001

^1^Odds Ratio

^2^95% Confidence Interval

*whereby medical costs—including pharmaceutical co-payments, are capped after reaching an annual threshold

**Table 5:** Variables independently associated with suboptimal adherence.

Covariate		Adjusted odds ratio	95% CI	P-value
Born in Australia	No			
	Yes	2.4	1.2–4.9	0.014
Not in a relationship	No			
	Yes	3.3	1.5–7.3	0.004
Medicare Safety Net* reached in previous 12 months	No			
	Yes	2.2	1.1–4.5	0.024
Living in subsidized housing	No			
	Yes	2.5	1.0–6.2	0.045
Receiving home care services	No			
	Yes	4.4	1.0–18.8	0.046
Linked to HIV community organisation(s)	No			
	Yes	2.4	1.1–5.4	0.033
Starting ART following patient request	No			
	Yes	3.0	1.3–7.0	0.012

*whereby medical costs—including pharmaceutical co-payments, are capped after reaching an annual threshold

**Table 6:** Adherence according to number of independent variables present.

Number of independent variables* present	n (%)	ART non-adherent	ART adherent	Odds Ratio (95%CI)	P-value**
		n (%)	n (%)		
0	47 (9.0)	4 (8.5)	43 (91.5)	1.0	-
1	163 (31.2)	14 (8.6)	149 (91.4)	1.0 (0.3–3.2)	0.987
2	194 (37.2)	25 (12.9)	169 (87.1)	1.6 (0.5–4.8)	0.408
3	91 (17.4)	26 (28.6)	65 (71.4)	4.3 (1.4–13.2)	0.007
4–7	27 (5.2)	9 (33.3)	18 (66.7)	5.4 (1.5–19.7)	0.007

*As listed in Table 5

** P-trend <0.0001

### Sensitivity analyses

Sensitivity analysis using backward-step and forward-step approaches yielded similar results (Table 7). In sensitivity analysis, we examined the 38 participants on twice-daily ART, and following removal of 23 participants receiving twice-daily ART who missed less than 2 doses per month within the previous three months from the suboptimal group (thereby reclassifying them to the adherent group, but maintaining the 15 participants on twice-daily ART with more than two-missed doses per month within the previous three months as non-adherent), three predictor variables remained statistically significant (reaching the Medicare safety net, receiving home care services, and self-requesting ART); the other four predictors in the primary model were no longer statistically significant (relationship status, country of birth, subsidised housing, and accessing HIV community organisations). One variable became of borderline significance: injecting crystal methamphetamines (AOR 23.8; 95%CI 1.1–524.4; p = 0.045). Of these 23 participants, 11 were prescribed a once-daily ART co-formulated nucleoside backbone (either tenofovir with emtricitabine or abacavir with lamivudine).

**Table 7:** Sensitivity analysis.

Risk Factor	≥3 (n = 78)	<3 (n = 444)	n =	Total	%	Univariate			Regression—Forward Step Wald			Regression—Enter Method			Regression—Backward Wald		
						OR	95% CI	p	AOR	95% CI	p	AOR	95% CI	p	AOR	95% CI	p
Currently not in a relationship																	
No	17	207	224		7.6												
Yes	61	237	298	522	20.5	3.1	1.8–5.5	<0.001	2.6	1.4–5.0	0.002	3.3	1.5–7.3	0.004	3.0	1.4–6.5	0.006
Born in Australia																	
No	15	186	201		7.5												
Yes	63	258	321	522	19.6	3.0	1.7–5.5	<0.001	2.7	1.4–5.1	0.003	2.4	1.2–4.9	0.014	2.3	1.1–4.7	0.019
Medicare Safety Net reached in previous 12 months																	
No	49	343	392		12.5												
Yes	29	101	130	522	22.3	2.1	1.2–3.7	0.009	2.1	1.2–3.9	0.013	2.2	1.1–4.5	0.024	2.2	1.1–4.4	0.022
Major Depressive Disorder (PHQ-9)																	
No	49	386	435		11.3												
Yes	29	58	87	522	33.3	3.9	2.3–6.7	<0.001	2.1	1.1–3.9	0.025						
Marijuana use ≥monthly																	
No	52	376	428		12.1												
Yes	26	68	94	522	27.7	2.8	1.6–4.7	<0.001	1.9	1.0–3.5	0.044						
Receives home care services																	
No	70	438	508		13.8												
Yes	8	6	14	522	57.1	8.3	2.8–24.8	<0.001	5.0	1.5–16.5	0.009	4.4	1.0–18.8	0.046			
Participates in HIV Community Organisations																	
No	56	389	445		12.6												
Yes	22	55	77	522	28.6	2.8	1.6–4.9	<0.001	2.3	1.2–4.4	0.012	2.4	1.1–5.4	0.033	2.3	1.1–5.0	0.032
Necessity Concerns score > sample mean																	
No	30	247	277		10.8												
Yes	48	197	245	522	19.6	2.0	1.2–3.3	0.005	1.8	1.0–3.1	0.050						
Starting ART following patient request																	
No	61	397	458		13.3												
Yes	17	47	64	522	26.6	2.3	1.3–4.3	0.006	2.7	1.3–5.5	0.005	3.0	1.3–7.0	0.012	3.0	1.3–6.9	0.008
Living in subsidized housing																	
No	56	362	418		13.4												
Yes	22	82	104	522	21.2	1.7	1.0–3.0	0.047				2.5	1.0–6.2	0.045	2.7	1.1–6.3	0.023
Having difficulty with ART pharmacy access																	
No	67	423	490		13.7												
Yes	11	21	32	522	34.4	3.3	1.5–7.2	0.001							2.8	1.0–7.6	0.050

Notable variables that were significant in bivariate, but not multivariate, analysis included ART-specific factors (non-single-tablet regimens, greater than once-daily ART dosing), injection drug use, and other markers of socioeconomic status (notably low income, unemployment, financial strain and cost barriers to ART access).

## Discussion

In our cohort of adults with HIV in Australia, with a median of three years of sustained viral suppression, 15% self-reported suboptimal ART adherence over the previous three months at a level that is likely to place them at increased risk of ART failure.

Other studies of self-reported ART adherence found similar or higher findings of suboptimal adherence. The average rate of reporting ≥90% adherence is estimated to be 62% [9]. One anonymous, community-based online survey (HIV Futures 7) found that about 20% of 1,058 HIV-positive, Australian adults reported <90% adherence [48]. While viral suppression in Australia is estimated to be 92% of those engaged in care; this represents only 68% of those diagnosed [10], it is unknown how much of that gap is represented by non-adherence.

We found several factors to be independently associated with suboptimal ART adherence, including socioeconomic, cultural, social, relationship, and patient engagement factors, but not clinical or ART regimen-related factors. Of these, 3 variables were common to all analyses: reaching the Medicare safety net, receiving home care services, and self-requesting ART.

Being born in Australia was associated with less adherence in our sample, which is supported by a higher rate of loss to follow-up in Australian-born individuals within another Australian cohort [49]; adherence data is not collected in that cohort. Mills et al [50] showed significantly higher adherence to ART in sub-Saharan Africa (77%) compared to North America (55%). Ortego et al [9] in meta-analysis found that participants in countries with high Human Development Index (HDI) had lower adherence rates than those with low HDI. However, in our sample, of those not born in Australia, the country of birth was most likely to be in the United Kingdom or New Zealand, where national healthcare is similarly available. In our cohort, there was a higher proportion of low-income earners in those who were Australian born, and a higher proportion of women in those born overseas; while other characteristics between the groups were similar.

Not being in a relationship was also independently associated with lower ART adherence. Other research has found adherence levels to increase with perceived quality of the relationship, and also with a partner’s support of ART [51].

About 20% of our cohort initiated ART with the aim of preventing transmission to others, and 12% at his/her own request. Therefore, it is noteworthy that suboptimal ART adherence was more common if the primary reason for having started ART was the patient’s own request. This is important, as the purpose of initiating ART, for personal or public health benefit, may influence a patient’s likelihood for adherence. Recently, in a study of HIV serodiscordant couples investigating ART as prevention of HIV (HPTN 052), high levels of ART adherence by pill count and self-report were reported in those initiating ART as prevention [52]. Good mental health was the only factor significantly associated with optimal ART adherence. HPTN 052 provides the only prospective adherence data regarding predictors of ART adherence in a population who initiated ART treatment as a means for prevention of HIV transmission. The participants were under clinical trial conditions, and actively received adherence counselling throughout the study visits, whether the same high levels of adherence will be achieved in the real world is unknown. Moreover, the investigators defined high-level adherence as ≥80% of prescribed doses, which is a lower threshold than we examined. Assessment of adherence over time in those who started ART at their own request will be important to follow-up in our cohort. We are unable to explain why those participants who self-requested ART (seemingly motivated therefore to initiate ART) would be less adherent. It may be that patients self-requesting ART may be doing so for reasons such as onward transmission (e.g. pressure from sexual partners or media), or lack of education around ART requirements or side effects once started. Also, given that our sample is highly treatment-experienced, it is possible that some of the patients self-requesting ART did so after an ART break [4].

One financial indicator independently associated with suboptimal ART adherence in our sample was living in subsidized housing. In comparison, one systematic review of five studies found that medication adherence was positively associated with housing stability in HIV-infected adults [53]. However, living in subsidized housing, as assessed in our study, does not necessarily imply that the housing is unstable (e.g. transient). The implication that subsidised housing may not represent housing stability is important; as one study found that residents in long-term public housing had better ART adherence than those in short-term or transient housing situations (e.g. hostels or shelters) [54].

In our sample, reaching the Medicare safety net was positively associated with suboptimal adherence. Housing status and meeting Medicare safety net thresholds may reflect broader differences in socioeconomic status (SES). Lower SES has been associated with poorer treatment outcomes in HIV, although this has predominantly been investigated in the United States where subsidised healthcare was not universally available [16], while our sample overwhelmingly has access to subsidized healthcare, as in western Europe and Canada for example. Those patients who meet the Medicare safety net threshold are more likely to have multiple comorbidities, given that the threshold is met by service utilisation. It may be that exceeding the Medicare safety net thresholds places participants at such a high level of financial strain that ART delay or interruption follows. This would suggest that lowering the Medicare safety net threshold may improve ART adherence. While the ART co-payment costs to participants were not associated with suboptimal adherence, this is just one component of the financial burden that contributes to meeting the Medicare safety net threshold (thereby reduced once the threshold is met). Recently (01 October 2015), the ART co-payment was removed in the state of New South Wales. Other jurisdictions in Australia still apply a co-payment, while some do not (or never have). The influence this has on ART adherence in different jurisdictions and over time is yet to be determined.

Suboptimal adherence also associated with home-care services and linkage to HIV-community organisations / outreach. These covariates may also be indicative of other factors that exacerbate non-adherence, as they may be the result of a higher dependency on medical care, or self-identified requirements to engage in outreach, for example, patients with chronic or more complex needs. These variables are unlikely to be the cause of low adherence per se, but rather may be a result of complex sets of factors that predispose to low adherence, such as low income, higher healthcare costs, low education, drug use, and mental ill-health.

Sensitivity analysis was conducted, fitting the model after reclassifying 23 participants from the “sub-optimally adherent” group as they were on twice-daily ART dosing. The two analyses overlapped in that statistically significant variables in the reduced sample were reaching the Medicare safety net, receiving home care services, and self-requesting ART; and of borderline statistical significance was injecting crystal methamphetamine. Importantly, 11 of these 23 participants were on a once-daily backbone therapy. It is unknown from our data which / what ART dose was missed.

Our study looked at a comprehensive set of factors that may influence adherence. In multivariate analysis, ART medications and regimens (e.g. STRs) were not as important as socioeconomic, cultural, social indicators or participant preference to start ART. Other factors, such as injection drug use and depression, were also no longer significant following regression analysis. Also contrary to previous work in the US which concentrated on more advanced HIV-positive cases with a higher prevalence of HIV-associated dementia [26], we did not find an association with neurocognitive impairment.

Our data suggest that improvements in ART dosing and tolerability may have less effect on improving adherence than will addressing the modifiable variables we found to be associated with suboptimal adherence. The findings of the current study arguably suggest that interventions designed specifically for this vulnerable part of the Australian population are further needed as, despite current supports, it is possible that complex and combined life stresses associated with lower socio-economic status in high-income countries lead to non-optimal adherence and so the risk of disease progression.

Our study has limitations. Adherence was based solely on participant self-report; which may overestimate true adherence [55]. Hence it is possible that suboptimal adherence is underestimated in this sample. It should be noted, however, that all participants had achieved sustained viral suppression. Our study did not collect refusal rates during recruitment due to the nature of establishing a multi-site clinical cohort, relying on divergent local site management. This limitation is of particular importance to the risk of selection bias, if there are a subset of patients who were considered ineligible by the sites or themselves unwilling to complete the questionnaire (e.g. given the length of time involved).

Findings may not generalise to other population groups, such as women and heterosexual men, to patients who are experiencing virological failure, or to other settings, such as one without access to free or highly subsidized ART, community supports or social housing. The sample and setting of this study nevertheless captures the community most affected by HIV in Australia [56] and it is reassuring that our cohort’s demographics are very similar to the national demographic data [57].

There is a risk that in such a comprehensive data-set, there will be co-linearity between some variables. We chose to use a multivariable regression model, as the aim was to identify variables that could be easily identified in a clinical consult, to enable healthcare workers to determine allocation of adherence-improving resources in a clinically meaningful way; and for this reason using multivariable regression modelling and conducting sensitivity analyses was the approach chosen. Other studies investigating correlates of adherence have used similar approaches [58].

In the current era of early and lifelong ART, it is imperative that a patient’s likelihood for suboptimal adherence be assessed at regular intervals, and that those who would benefit from adherence interventions are identified and offered appropriate supports. From a broadly scoped, multi-dimensional assessment we identified a limited number of variables independently associated with suboptimal adherence. These variables are not routinely monitored in clinical care, cohort studies or clinical trials; but could be incorporated into these settings. These variables provide information that could be readily assessed prior to ART initiation and periodically whilst receiving ART as indicators of an elevated risk of suboptimal adherence. It will be important to undertake a longitudinal assessment of associations between covariates and suboptimal adherence, drawing on the data prospectively collected in the current study, to gauge if the predictors of incident and sustained low adherence are similar to covariates identified in the current report.

## Supporting information

S1 File Minimal data set. (SAV)
